# Adipokines in Neuroendocrine Tumors: An Evaluation of the Serum Levels of Ghrelin and Leptin

**DOI:** 10.3390/ijms25189820

**Published:** 2024-09-11

**Authors:** Janusz Strzelczyk, Agnes Bocian-Jastrzębska, Joanna Katarzyna Strzelczyk, Monika Wójcik-Giertuga, Krzysztof Biernacki, Dariusz Kajdaniuk, Beata Kos-Kudła

**Affiliations:** 1 Department of Endocrinology and Neuroendocrine Tumors, Department of Pathophysiology and Endocrinology, Faculty of Medical Sciences in Zabrze, Medical University of Silesia, 40-514 Katowice, Poland; bocian.agnes@gmail.com (A.B.-J.); monikawojcik90@gmail.com (M.W.-G.); beatakos@ka.onet.pl (B.K.-K.); 2 Department of Medical and Molecular Biology, Faculty of Medical Sciences in Zabrze, Medical University of Silesia, 41-808 Katowice, Poland; jstrzelczyk@sum.edu.pl (J.K.S.); kbiernacki@sum.edu.pl (K.B.); 3 Department of Pathophysiology and Endocrinology, Faculty of Medical Sciences in Zabrze, Medical University of Silesia, 41-808 Katowice, Poland; dkajdaniuk@sum.edu.pl

**Keywords:** neuroendocrine tumors, adipokines, ghrelin, leptin, cachexia

## Abstract

Neuroendocrine tumors (NETs) are a heterogeneous group of tumors that are characteristically different from other malignancies. The difference is not only in the prognosis, which is usually more favorable in such patients, but also in the high clinical progression of the disease, where NET patients do not experience the cachexia typical of other malignancies. The purposes of this study were to evaluate the ghrelin and leptin levels in a group of patients diagnosed with gastroenteropancreatic neuroendocrine tumors (GEP-NETs) and bronchopulmonary neuroendocrine tumors (BP-NETs) and to analyze the relationship between the body mass index (BMI), cachexia and selected NET markers. The study group comprised 52 patients with GEP-NETs and BP-NETs, while the controls comprised 67 healthy volunteers. The ghrelin and leptin concentrations were determined in both groups. The concentrations of chromogranin A, serotonin, 5-hydroxyindoleacetic acid (5-HIAA), total cholesterol, triglycerides and glucose were determined in the study group. Characteristics of the study group and of the controls were defined by age, sex and BMI, and the effects of these factors on the ghrelin and leptin concentrations were assessed. The data obtained were subject to statistical analysis. The study cohort showed higher levels of ghrelin as compared to the controls (142.31 ± 26.00 vs. 121.49 ± 35.45, *p* = 0.016), and no statistical difference in the levels of leptin (11.15 ± 9.6 vs. 12.94 ± 20.30, *p* = 0.439) were observed. Significantly lower levels of leptin were found in patients with the small intestine primary location, as compared to individuals with primary locations in the lungs and the pancreas (4.9 ± 6.49 vs. 16.97 ± 15.76, *p* = 0.045, and 4.9 ± 6.49 vs. 12.89 ± 8.56, *p* = 0.016, respectively). A positive correlation was observed between the leptin levels and the BMIs in both the study group (r_S_ = 0.33, *p* = 0.016) and the controls (r_S_ = 0.41, *p* = 0.001). The study group showed a negative correlation between the leptin levels and 5-HIAA (r_S_ = −0.32, *p* = 0.026) and a negative correlation between the leptin levels and Ki-67 (r_S_ = −0.33, *p* = 0.018). The control group showed negative correlations between the ghrelin and the volunteer age (r_S_ = −0.41, *p* = 0.008), the leptin and the volunteer age (r_S_ = −0.44, *p* < 0.001), the leptin and total cholesterol (r_S_ = −0.24, *p* < 0.049) as well as the leptin and triglycerides (r_S_ = −0.33, *p* < 0.006). The current study emphasized the importance of the markers’ determination, where ghrelin appears as a valuable diagnostic biomarker in NETs, probably responsible for maintaining a normal BMI, despite the progression of the disease.

## 1. Introduction

Neuroendocrine tumors (NETs) are a heterogeneous group of tumors of different localizations that originate from cells of the diffuse endocrine system (DES). The incidence of such tumors is increasing [1,2]. Gastroenteropancreatic neuroendocrine tumors (GEP-NETs) with gastroenteropancreatic localization are the most commonly diagnosed, and the respiratory system (bronchopulmonary neuroendocrine tumors (BP-NETs)) appears to be the second most common location [1]. NETs are capable of secreting multiple hormones, manifesting clinically as typical carcinoid syndrome. However, the tumors may fail to produce sufficient hormones, remain hormonally inactive and develop in secret for many years before they are eventually diagnosed at the stage of significant dissemination, which is the most common case [1]. According to the eighth edition of the AJCC/UICC (American Joint Committee on Cancer/Union for International Cancer Control) recommendations, the ESMO (European Society for Medical Oncology) guidelines [2] and the 2022 WHO (World Health Organization) classification [3], NETs are defined as NET G1 (Ki-67 < 3%), NET G2 (Ki-67 3–20%), NET G3 (Ki-67 > 20%, usually between 21–55%) and NEC (neuroendocrine carcinoma, Ki-67 usually >55%) (i.e., low-differentiated carcinomas with a highly aggressive clinical course) [1,2,3]. A unique feature of this group of cancers is the very rare or absent cachexia, typical of advanced cancers, even at the stage of massive progression [4].

For many years, the adipose tissue was considered to be merely an energy store. Nowadays, it is recognized as a specific endocrine organ that produces multiple biologically active substances called adipokines or adipocytokines. Ghrelin is a 28-amino acid peptide hormone that was discovered in 1999 by Kojima et al. [5]. Most of the blood ghrelin is released from the lining cells (the so-called X/A-like cells) of the body and the fundus of the stomach. The hormone exists in two forms: the inactive deacylated form and the active acylated form, synthesized under the influence of ghrelin-O-acyl-transferase (GOAT) [6,7]. Ghrelin has been recognized as the “hunger hormone”, as its primary role is to stimulate the appetite through the activation of the neuropeptide Y (NPY) neurons in the hypothalamus, thereby enhancing energy intake [5,8,9].

Leptin is also a peptide regulatory hormone for fat metabolism, also classified as an adipokine; however, contrary to ghrelin, leptin is called the “satiety hormone”. The adequate bodily function of leptin helps to maintain a proper body weight. Disorders of both the leptin function and its receptor function may result in obesity, as confirmed by numerous studies. Leptin can also be used as a medication, e.g., in lipodystrophy-a disorder associated with the local or generalized atrophy of the fat mass, where reduced concentrations of this hormone are found [10,11].

Both ghrelin and leptin are adipokines showing pleiotropic action and have broad effects on multiple mechanisms of the body homeostasis [8,9]. The regulatory function of adipokines in energy homeostasis has already been well established, and more and more scientific reports have now pointed to their role in oncogenesis. Among the mechanisms of the tumor growth and progression is imbalanced adipokine secretion [12,13,14]. The purposes of the present study were to evaluate the serum concentrations of both ghrelin and leptin in a group of NET patients and to investigate the relation between the BMI and the selected NET markers, as well as the total cholesterol, triglyceride and glucose levels.

## 2. Results

### 2.1. The Study and Control Cohorts

The observed levels of ghrelin were statistically higher in the study group than in the controls (142.31 ± 26.00 vs. 121.49 ± 35.45, *p* = 0.016). The levels of leptin were not significantly different between the study group and the controls (11.15 ± 9.60 vs. 12.94 ± 20.3, *p* = 0.439). The study group showed significantly higher levels of glucose than those of the controls (92.39 ± 18.74 vs. 84.94 ± 7.89, *p* = 0.019), and the levels of triglycerides were significantly lower than those of the controls (1.40 ± 0.65 vs. 1.49 ± 0.29, *p* = 0.006).

### 2.2. Ghrelin

We observed no significant difference in the ghrelin levels as related to sex, tumor type, primary tumor location site, TNM (TNM Classification of Malignant Tumors), histological grading, Ki-67 or the presence of metastasis or the metastasis location in the study group.

The study group showed no correlations between ghrelin and leptin and any of the demographic (age, BMI), biochemical (chromogranin A, serotonin, 5-HIAA, glucose, total cholesterol, triglycerides) or clinical (TNM, histological grade or Ki-67) parameters. The control group showed a negative correlation with the participant age (r_S_ = −0.41, *p* < 0.008).

### 2.3. Leptin

**Figure 1:**
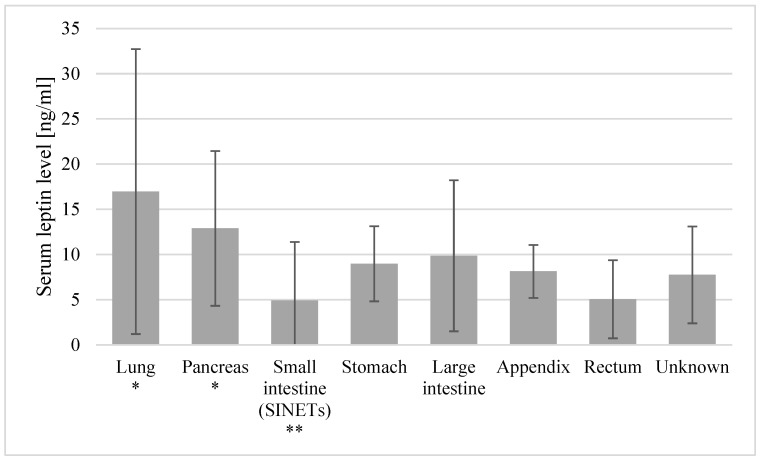
Mean leptin levels with standard deviations in patients with various primary site locations. * Locations with leptin levels significantly higher than the small intestine (SINET) levels. ** Locations with leptin levels significantly lower than those in lung and pancreas primary tumors.

The mean leptin levels were significantly higher in the female patients as compared to the males (14.4 ± 10.5 vs. 5.4 ± 3.7, *p* < 0.001), with the tendency reversed in the control group, where the observed leptin levels were lower in the female volunteers than in the males (10.95 ± 6.6 vs. 14.6 ± 27.6, *p* = 0.01). The patients with the primary tumor location within the small intestine (SINETs) showed lower leptin levels than those of patients with other tumor locations (4.9 ± 6.49 vs. 11.97 ± 9.7, *p* = 0.018). Significantly lower levels of leptin were observed in patients with tumors located in the small intestine (SINETs) as compared to patients with tumors located in the lungs (4.9 ± 6.49 vs. 16.97 ± 15.76, *p* = 0.045) and the pancreas (4.9 ± 6.49 vs. 12.89 ± 8.56, *p* = 0.016). No other primary site locations showed any significant differences in the leptin levels (Figure 1). No significant differences in the leptin levels were found between the BP-NETs and GEP-NETs, nor between patients with various histological grades, TNM stages, Ki-67 classes and locations of metastasis.

Positive correlations between the leptin levels and BMI were also observed in both the study group (r_S_ = 0.33, *p* = 0.016) and the controls (r_S_ = 0.41, *p* = 0.001). Also, the study group showed a negative correlation between the leptin levels and 5-HIAA (r_S_ = −0.32, *p* = 0.026) and a negative correlation between the leptin levels and Ki-67 (r_S_ = −0.33, *p* = 0.018). Moreover, it was only in the control group that we observed a negative correlation between the leptin levels and the healthy volunteer age (r_S_ = −0.44, *p* < 0.001), between the leptin levels and the total cholesterol (r_S_ = −0.24, *p* < 0.049) and between the leptin levels and triglycerides (r_S_ = −0.33, *p* < 0.006). No other correlations between the leptin and other biochemical parameters were observed in the study or the control group. Also, no other correlations between leptin and the clinical parameters (TNM, histological grade) were observed in the study group.

### 2.4. Other Demographic, Clinical and Biochemical Parameters in the Study Group

We observed that the mean BMI was lower in patients with the tumor located in the appendix than that in other locations (20.05 ± 3.5 vs. 25.3 ± 3.5, *p* = 0.045), and it was lower in patients with T = 3 than in other patients (21.7 ± 2.3 vs. 25.4 ± 3.7, *p* = 0.02). The triglyceride levels in the patients with tumors located in the appendix were lower than those in other patients (0.8 ± 0.3 vs. 1.4 ± 0.7, *p* = 0.049).

The chromogranin A levels were higher in patients with confirmed metastasis than in those with no detectable metastasis (173.0 ± 292.0 vs. 24.3 ± 29.6, *p* = 0.001).

The 5-HIAA mean levels were significantly lower in the female patients than in the males (11.7 ± 18.9 vs. 16.7 ± 27.2, *p* = 0.028) and significantly higher in the GEP-NET patients as compared to the BP-NET patients (15.2 ± 24.1 vs. 5.6 ± 3.4, *p* = 0.018), and the patients with confirmed metastasis also showed higher levels of 5-HIAA than patients without metastasis (19.5 ± 29.5 vs. 7.0 ± 3.4, *p* = 0.011). The T = 3 patients showed lower 5-HIAA levels than those of the other patients (4.2 ± 1.7 vs. 14.3 ± 23.4, *p* = 0.006).

## 3. Discussion

As a peripheral orexigenic factor, ghrelin is directly linked to energy balance control, stimulating appetite, initiating food intake and inhibiting adipocyte apoptosis [15]. In addition to its role as the bodily energy and weight regulator, it has been described as being involved in multiple pathological conditions [8,9,15]. The presence of the growth hormone secretagogue receptors (GHS-Rs), known as ghrelin receptors, has been identified in the endocrine organs and on the surfaces of immune system cells and cancer cells, including gastrointestinal stromal tumors [15,16,17,18,19]. It has been shown that in conditions such as inflammatory bowel disease, rheumatoid arthritis and pancreatitis, the ghrelin levels are significantly elevated, along with the severity of the disease, and they are inversely related to the expression of the pro-inflammatory cytokines. In animal models, the supply of ghrelin induced the suppression of the tumor necrosis factor alpha (TNFα), IL-6 and CRP (C-Reactive Protein) production and affected the prolonged survival [17]. Ghrelin appears to play an important part in oncological diseases. This orexigen hormone performs the essential role in some crucial processes of cancer progression, including cell proliferation, migration and invasion [17]. Ghrelin has also been shown to inhibit the tumor cell proliferation in various types of cancers [17]. Additionally, ghrelin may serve as a prognostic factor, with the potential role of ghrelin analogs in the treatment of cancer [20,21].

The expressions of some components of the ghrelin system in human NET tissues and in the adjacent healthy tissues have been found to be significantly different. Papotti et al. [22] revealed that the majority of gastric carcinoids and some intestinal endocrine tumors showed immunoreactivity for ghrelin. Grönberg et al. [23] demonstrated that the expressions of the peptide hormones ghrelin and obestatin were predominantly expressed in tumors of foregut and hindgut origin. Luque et al. [24] determined the expression levels of In1-ghrelin, GOAT and GHSR1a/1b receptors in the neuroendocrine tumor. The expression levels of these key ghrelin system components were elevated and positively correlated in the tumor tissues, compared to the normal/adjacent tissues, and were higher in patients with the progressive disease. Moreover, In1-ghrelin increased the aggressiveness of NET cells, affecting their proliferation and migration. As reported by the authors, this may allow for the use of some components of the ghrelin system as potential prognostic markers. According to Herrera-Martínez et al. [25], the evaluation of the GOAT activity also seems very promising. This is because the enzyme was significantly overexpressed based on a quantitative evaluation by PCR (Polymerase Chain Reaction) in GEP-NET tumor samples. The enzyme has also been shown to be directly correlated with the tumor diameter and may represent a new diagnostic biomarker for such patients. In another study [26], it was proved that the widespread expression of key somatostatin and ghrelin system components in lung carcinoids was associated with the clinical–histological features. Both functioning and non-functioning PanNETs express ghrelin in up to 95% of cases [27].

In the current study, we observed higher levels of ghrelin in the study group as compared to the controls. The literature on the association of serum ghrelin with NETs is scarce and conflicting. Adrichem et al. [28] showed no significant differences in the ghrelin levels between NET patients and the controls. Corbetta et al. [29] claim that carcinoids and pancreatic tumors rarely cause ghrelin hypersecretion. However, elevated ghrelin has also been found in NETs [30]. Other authors believe that the determination of ghrelin in patients with malignant gastric NETs is valuable [31]. A case of metastatic PanNETs, which presented as ghrelinoma and later transformed into symptomatic insulinoma, is an example indicating that changes in the functional biology of a tumor can sometimes be more pathogenic than the metastatic disease itself [32]. Moreover, the authors of [32] showed clearly that the presence of metastatic foci in the liver correlated positively with increased ghrelin levels, compared to patients without liver metastases, and the increase in the BMI was also associated with higher ghrelin levels in both subgroups [32]. Similarly, ghrelin levels correlated positively with the total area of the primary tumor as well as the area of the largest metastatic lesion in the liver. In addition, the study showed a negative correlation between the chromogranin A and the ghrelin levels in NET patients with liver metastases, while NET patients without liver metastases showed a slight positive correlation [33]. The authors conclude that an increased ghrelin-secreting tumor cell mass may play a role in overcoming the resistance, as usually observed in cachexia [33].

The tumor process is a specific systemic inflammation, sustained by cytokines responsible for hypercatabolism, the production of acute-phase proteins and cachexia [6]. Cachexia is defined as a multifactorial syndrome characterized by the loss of lean body mass with or without fat loss [34]. It results from the deterioration of the nutritional status and is responsible for as much as 30% of deaths among cancer patients. Decreased muscle and fat mass not only negatively affects patients’ quality of life but also worsens the prognosis, often by decreasing the effectiveness of the treatment used and increasing the number of complications [34]. The stimulatory factors produced by the tumor cause the activation of the inflammatory cascade, which activates the adrenal glands to release cortisol and methoxy-catecholamines and causes the catabolism of proteins, carbohydrates and lipids. The indicators of inflammation and cytokines are kinds of biomarkers for cancer cachexia. Among these, ghrelin, adiponectin and leptin may be mentioned, along with IL-1, IL-6 and TNFα [34]. Obese people show reduced basal levels of ghrelin, while those affected by anorexia or cachexia have elevated levels [18].

Wolf et al. [35] determined the ghrelin mean plasma levels in 40 breast and colon cancer patients to show significantly elevated values in patients with cachexia. The authors believe that an increased ghrelin level is a compensatory effect of the weight loss, while cachexia is the result of the ghrelin resistance induced by high levels of pro-inflammatory cytokines. Partial ghrelin resistance, which occurs in patients with cancer cachexia, can be overcome by the exogenous ghrelin [36]. Such short-term use is safe and well tolerated, and in view of its additional anti-apoptotic and anti-inflammatory effects, it may appear as a new therapeutic option [33,37].

It has been extremely interesting to observe a common lack of cachexia, typical of other cancers, also in NET patients, even if the tumor process is significantly advanced [4,30]. This observation is concordant with our data. The study group patients with elevated ghrelin levels did not present cachexia, which confirms this phenomenon. One study hypothesized that elevated levels of the endogenous ghrelin produced by NET cells produces the orexigenic effect, thereby allowing patients’ BMIs to remain normal [33]. Another study also reported the importance of ghrelin in NETs, as well as its role in maintaining normal body weights in these patients, despite the clinical progression of the disease [38]. Ekablad et al. [39] indicated that the plasma ghrelin levels were significantly higher in patients with MEN1 syndrome tumors, as compared to the group of patients with sporadic tumors. Despite the large size of the tumor and numerous liver lesions, the BMIs of these patients did not differ from the parameters of the control group without any established neoplastic lesions. The group of NET patients showed no symptoms of cachexia. In one patient, radical surgical treatment of the pancreatic lesions resulted in a 42% decrease in their ghrelin levels and a 20% decrease in their BMI. The authors suggest that sufficiently high levels of ghrelin may prevent the development of cancer cachexia by the orexigenic mechanism that overcomes the anorexigenic effect induced by inflammation [39].

Furthermore, the control group showed a negative correlation between ghrelin and the participant age, which, however, was not confirmed in the study group. The observed decline in the ghrelin levels with age could be related to the weight loss and sarcopenia noted during physiological aging [40].

Also, in our study, ghrelin did not correlate with the biochemical or clinical parameters. Correlations between acylated ghrelin and age as well as between the acylated ghrelin/unacylated ghrelin ratio and chromogranin A levels were observed in another study including NET patients with primary locations in the small intestine, pancreas, stomach and lungs and those of unknown origin [28].

In their study, Soleyman-Jahi et al. [18] showed that the ghrelin levels could predict the survival of patients with gastric cancer, regardless of the stage and cachectic features. The better survival and prognoses for those with the highest concentrations of ghrelin were explained by the ghrelin’s mitogenic potential and the anti-inflammatory effect limiting tumor progression. Moreover, the orexigenic potential of the hormone itself may translate into an improved prognosis. After gastrectomy, in the microenvironment for paracrine, the mitogenic effect of ghrelin against the tumor cells is eliminated and the anti-inflammatory and appetite-stimulating components predominate, thereby improving survival [18]. An important conclusion is the influence of the characteristics of the given type of tumor on the resultant effect of ghrelin. Unfortunately, due to the lacking data, we were unable to assess the correlation between ghrelin and the outcome.

Known also as the “satiety hormone”, leptin is an adipokine produced by white adipose tissue, whose overarching role is to regulate the energy balance through a central anorexigenic effect. The plasma concentration of this adipokine correlates with the fat mass and, according to some authors, is ten times higher in obese individuals [41]. In our study, in both analyzed groups, the leptin levels correlated positively with the BMI. The highest leptin concentrations were presented by obese patients, while the lowest values were observed in the underweight patients. The results obtained are consistent with the literature confirming higher leptin concentrations in obese individuals [41]. Peripherally, leptin has a role in reproduction, hematopoiesis, glycemic regulation and inflammation, as well as in the development of many different malignancies [12]. Leptin leads to the proliferation of cancer cells through the activations of the JAK/STAT (Janus kinaze/signal transducer and activator of transcription), MAPK/ERK (mitogen activated protein kinase/extracellular-signal-regulated kinase), PI3K/Akt (phosphatidylinositol 3-kinase/protein kinase B) and suppressor cytokine signaling pathways [42,43]. It was demonstrated that leptin receptors were plentiful in many types of cancer and that leptin–leptin receptor signaling played a key role in the promotion of several processes involved in cancer progression, such as the epithelial–mesenchymal transition, angiogenesis, metastasis and chemoresistance [12,13,42,44,45]. Leptin acts as a tissue growth factor and angiogenesis-stimulating factor, comparable to the vascular endothelial growth factor (VEGF) [14]. Moreover, it induces cyclooxygenase-2 (COX-2) expression, which directly affects progression and the metastatic potential [46]. Potentially, leptin can be used as a marker to monitor the course of the treatment [47]. In our study, the levels of leptin between the study group and the controls were not significantly different. In addition, we observed no significant differences between the leptin levels and the different locations of metastasis. Nevertheless, to the best of our knowledge, the current study is the first to demonstrate in NETs that the primary site locations show significant differences in their leptin levels. Tissue-specific effects of leptin are therefore possible. We observed significantly lower levels of leptin in patients with the small intestine primary location, as compared to patients with the examined primary sites, especially the lungs and pancreas (Figure 1). The intestinal epithelial cells promote the secretion of leptin [48]. Several reports have been published on the autocrine and endocrine functions of leptin as a trophic factor [49,50,51]. Leptin deficiency changes the functioning of the intestinal cells [52] and impairs both cell proliferation and cell death [53]. Also, higher leptin concentrations in the pancreatic and pulmonary locations may indicate different roles of leptin in the cancer pathogenesis and its involvement in cancer-linked systemic inflammation.

The results of a study evaluating the leptin levels in PanNETs show that patients with a distal form of the disease have lower leptin levels [54]. In the current study, the different locations of metastasis did not differ in their leptin levels.

Similar to the authors of [54], we observed negative correlations between leptin and Ki-67, as well as between leptin and 5-HIAA. Our results showed that the leptin levels were lower along with increasing Ki-67 values and 5-IHAA concentrations. This correlation is a potential link to NETs and leptin determination and may be useful in the accompanying determination of the known NET markers.

In the states of cachexia, the leptin levels are reduced and correlate inversely with the levels of pro-inflammatory cytokines [14]. It is suggested that, in malnutrition associated with cancer, leptin is not only an exponent of changes in the fat mass but is also an acute-phase reactant. Cytokines may act as leptin-like factors to inhibit food delivery and increase the consumption of energy substrates. Low leptin levels in cachectic patients may also result from the secretion of the leptin-suppressing substance in advanced cancer [55]. As mentioned above, the current research involved patients who were not cachectic, and the leptin levels did not differ between the assessed groups. In the control group, leptin negatively correlated with the volunteers’ ages, total cholesterol and triglycerides. Unlike the controls, in the study group, the mean leptin levels were significantly higher in the female patients than in the males. Numerous studies confirm a significant sex dimorphism in leptin production. Compared to males, female rodents have been shown to secrete two to four times more leptin. Female leptin levels have not been shown to be dependent on age, the gonadal hormonal function or the hormone replacement therapy used [56]. Rosiek at al. [57] noted higher levels of cholesterol, triglycerides and glucose in PanNET patients with BMIs ≥ 25 kg/m^2^. Santos et al. [58] made it known that well-differentiated GEP-NETs are related to elevated triglycerides. In our study, the triglyceride levels and BMIs in the patients with tumors located in the appendix were lower than those in other patients. Decreased triglyceride levels result from and are associated with a lower BMI [59]. Potentially, this could indicate that this location increases the risk of cachexia. Metabolic disturbances coexist with NETs and affect both their occurrence and course.

5-HIAA is biochemical marker in patients with carcinoid syndrome, and gender-related differences in these patients were reported in an Italian multicenter cohort study, which showed that carcinoid syndrome was slightly more frequent in males than females, which influenced the clinical presentation but not the type of treatment or progression-free survival (PFS) [60]. In the current study, carcinoid syndrome was not present in the patients.

## 4. Materials and Methods

**Figure 2:**
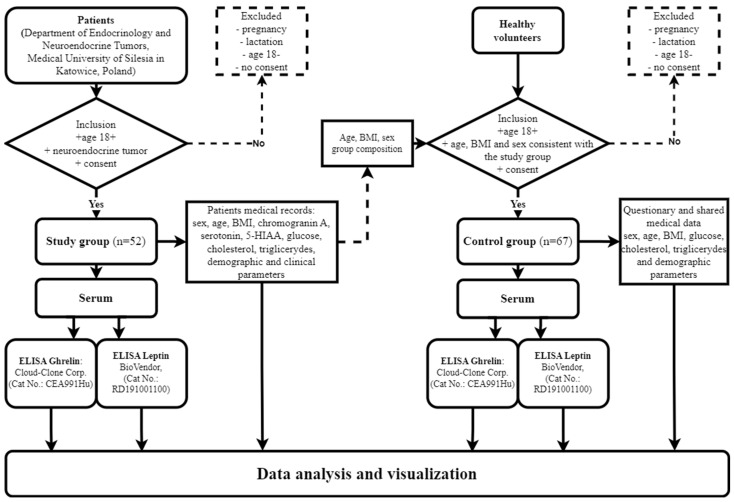
Workflow demonstrating inclusion and exclusion of patients and healthy volunteers in study and control groups.

The study observed the principles of Good Clinical Practice and the Declaration of Helsinki and was approved by the Ethics Committee of the Medical University of Silesia, Poland (KNW/022/KB1/137/I/14 and PCN/CBN/0052/KB1/24/II/22). All the patients were recruited from the Department of Endocrinology and Neuroendocrine Tumors, Medical University of Silesia in Katowice, Poland, and their complete medical histories were taken. The main inclusion criteria for the study group were the diagnosis of the neuroendocrine tumor, an age above 18 and the signed informed consent to participate in the study. The study group included patients in whom the treatment had not yet commenced. The exclusion criteria for both groups were pregnancy, lactation, an age below 18 and the failure to deliver the informed consent to participate in the study. Following the consent obtained from the participants, peripheral blood samples were taken and placed into S-Monovette tubes with a clotting activator (Sarstedt, Germany), and serum was obtained from the collected blood (centrifuged at 1500 rpm for 15 min). The serum samples were frozen down to −80 °C for further assessment of the ghrelin and leptin concentrations, making use of the ELISA method. Data concerning patients’ neuroendocrine markers, including chromogranin A, serotonin and serotonin metabolites and 5-hydroxyindole acetic acid (5-HIAA), were retrieved from medical records. The above markers are determined on a routine basis regardless of the presence of carcinoid syndrome. The influences of drugs, diet, renal dysfunction and other co-morbidities on the determination of the markers were excluded. The non-modifiable elements (age, sex) were analyzed along with the modified ones; the body weight was defined as the BMI, and the body weight fluctuations were included in the objective recognition. The workflow demonstrating the inclusion and exclusion of patients and healthy volunteers in the study is presented as a flowchart (Figure 2).

### 4.1. The Study Group and the Controls

The study cohort consisted of 52 patients (33 women and 19 men) with a mean age of 55.33 ± 11.11 years and mean BMI of 24.99 ± 3.66, 43 patients (82.69%) with neuroendocrine tumors of the digestive tract (GEP-NETs) and 9 patients (17.31%) with respiratory system tumors (BP-NETs). BP-NETs are classified by four histological variants: typical carcinoma (TC), atypical carcinoma (AC), large-cell neuroendocrine carcinoma (LCNEC) and small-cell lung cancer (SCLC). Carcinoid syndrome was not present in the patients. The hospital records were verified to provide information about the demographic and clinical parameters of each of the patients, including the primary tumor location, the grade, the clinical stage (TNM staging), the Ki-67 proliferation index as well as details of the metastasis at the sampling time, as illustrated in Table 1. Detailed demographic and biochemical data are presented in Table 2. The diagnostic criteria for cachexia obtained in the study were as follows: a 5% weight loss in 12 months and the presence of asthenia and albumin concentrations below 3.2 g/dL.

The healthy controls included 67 individuals (31 women and 36 men) with a mean age of 54.99 ± 3.59 years and mean BMI of 24.02 ± 2.69. The demographic data were acquired from questionnaires, and leptin and ghrelin level measurements were performed in both groups (52 patients and 67 healthy individuals). The control group summary is presented in Table 2. The levels of chromogranin A, serotonin and 5-HIAA were not measured in the control group. The control group was age-, sex- and BMI-matched with the study group.

### 4.2. Enzyme-Linked Immunosorbent Assay (ELISA)

Ghrelin and leptin were assayed in serum samples using ELISAs, according to the manufacturers’ recommendations: Cloud-Clone Corp., Houston, TX, USA (Cat No. CEA991Hu) and BioVendor, Brno, Czech Republic (Cat No. RD191001100), respectively. All the serum samples and standards were run in a doublet. A Universal Microplate Spectrophotometer, set to 450 nm as the primary wavelength (Bio-Tek µQuant, Bio-Tek, Winooski, VT, USA), was used to determine the absorbance of each well of the ELISA plates. Data Analysis Software KCJunior version 1.41.8 (Bio-Tek, Winooski, VT, USA) was applied, and the results were reported as the concentrations of ghrelin (pg/mL) and leptin (ng/mL) in the serum samples.

### 4.3. Statistical Analysis

The levels of the biochemical and demographic parameters in the study group vs. the controls and within the study group were compared making use of the Kruskal–Wallis test. The Spearman rank correlation coefficient (r_S_) was used to assess the correlation of both the biochemical and demographic parameters. All the data were presented as numbers of cases (n) and mean values ± standard deviations (SDs). The value of *p* < 0.05 was considered statistically significant. Data analysis and visualization were performed using R version 4.3.1 in RStudio version 2023.06.1 build 524 (PBC, Boston, MA, USA) using the stats R library [61] and Microsoft Excel version 2108 (14332.20503) (Microsoft, Redmond, WA, USA).

## 5. Conclusions

The identification of the diagnostic and prognostic markers and a better understanding of NET biology are necessary steps towards improved prognoses for cancer patients. Evaluating the adipokine concentrations in such patients, previous studies have offered promising results towards the use of these parameters in monitoring the clinical course of the disease. The current study emphasizes the importance of the determination of these markers. In our opinion, ghrelin seems to be a valuable diagnostic biomarker in NETs and is likely responsible for maintaining a normal BMI, despite the stage of the disease. In view of the results obtained, further studies on the involvement of adipokines in the pathogeneses of GEP-NETs and BP-NETs are warranted.

### Limitations

The study is limited by the small and heterogeneous patient population, the limited statistical power and the inability to perform extensive subgroup analysis. Further studies on expanding this research that are well balanced with regard to sex, age and tumor location populations are required.

## Figures and Tables

**Table 1:** Characteristics of the study group.

			n (% of Patients)
BP-NET cases 9 (17.31%)	Location of the primary tumor	Lung	9 (17.31%)
	Histological grade	Typical carcinoid (TC)	6 (11.54%)
		Atypical (ATC)	3 (5.77%)
GEP-NET cases 43 (82.69%)	Location of the primary tumor	Pancreas	20 (38.46%)
		Small intestine (SINETs)	6 (11.54%)
		Stomach	5 (9.62%)
		Large intestine	3 (5.77%)
		Appendix	3 (5.77%)
		Rectum	2 (3.85%)
		Unknown	4 (7.69%)
	Histological grade	NET G1	34 (65.38%)
		NET G2	4 (7.69%)
		NET G3	5 (9.63%)
T	T1		26 (50.0%)
	T2		16 (30.77%)
	T3		6 (11.54%)
	T4		2 (3.85%)
	Undetermined		2 (3.85%)
N	N0		37 (71.15%)
	N1		15 (28.85%)
M	M0		23 (44.23%)
	M1		29 (55.77%)
Ki-67	<20%		44 (84.62%) (6 BP-NET ATC + 38 GEP-NET)
	≥20% and <55%		8 (15.38%) (3 BP-NET ATC + 5 GEP-NET)
Metastasis	Total		27 (51.92%)
	Liver metastases		22 (42.31%)
	Lymph node metastases		12 (23.08%)
	Bone metastases		3 (5.77%)
	Other metastases		6 (11.54%)

**Table 2:** Summary of the demographic and biochemical characteristics of the study and control groups with *p*-values as delivered by the Kruskal–Wallis test.

Parameter	Study Group	Control Group	
	n (%)	n (%)	*p*
Sex [F: female, M: male]	F: 33 (63.46%) M: 19 (36.54%)	F: 31 (46.27%) M: 36 (53.73%)	0.067
	**Mean ± SD**	**Mean ± SD**	***p***
Age [years]	55.33 ± 11.11	54.99 ± 3.59	0.087
BMI [kg/m^2^]	24.99 ± 3.66	24.02 ± 2.69	0.073
Leptin [ng/mL]	11.15 ± 9.6	12.94 ± 20.30	0.439
Ghrelin [pg/mL]	142.31 ± 26.00	121.49 ± 35.45	0.016
Chromogranin A [µg/L]	95.55 ± 214.45	-	-
Serotonin [ng/mL]	257.13 ± 245.64	-	-
5-hydroxyindole acetic acid (5-HIAA) [mg/24 h]	13.51 ± 22.10	-	-
Glucose [mg/dL]	92.39 ± 18.74	84.94 ± 7.89	0.019
Total cholesterol [mmol/L]	4.97 ± 1.08	5.09 ± 0.65	0.889
Triglycerides [mmol/L]	1.40 ± 0.65	1.49 ± 0.29	0.006

## Data Availability

The original contributions presented in the study are included in the article, further inquiries can be directed to the corresponding author.
